# Rapid Turnover of the Cardiac L-Type Ca_V_1.2 Channel by Endocytic Recycling Regulates Its Cell Surface Availability

**DOI:** 10.1016/j.isci.2018.08.012

**Published:** 2018-08-16

**Authors:** Rachel Conrad, Gabriel Stölting, Johnny Hendriks, Giovanna Ruello, Daniel Kortzak, Nadine Jordan, Thomas Gensch, Patricia Hidalgo

**Affiliations:** 1 Institute of Complex Systems 4, Zelluläre Biophysik, Forschungszentrum Jülich, 52425 Jülich, Germany; 2 Institute of Biochemistry, Heinrich-Heine University, Düsseldorf, Germany

**Keywords:** Optical Imaging, Molecular Mechanism of Behavior, Cell Biology

## Abstract

Calcium entry through Ca_V_1.2 L-type calcium channels regulates cardiac contractility. Here, we study the impact of exocytic and post-endocytic trafficking on cell surface channel abundance in cardiomyocytes. Single-molecule localization and confocal microscopy reveal an intracellular Ca_V_1.2 pool tightly associated with microtubules from the perinuclear region to the cell periphery, and with actin filaments at the cell cortex. Channels newly inserted into the plasma membrane become internalized with an average time constant of 7.5 min and are sorted out to the Rab11a-recycling compartment. Ca_V_1.2 recycling suffices for maintaining stable L-type current amplitudes over 20 hr independent of *de novo* channel transport along microtubules. Disruption of the actin cytoskeleton re-routes Ca_V_1.2 from recycling toward lysosomal degradation. We identify endocytic recycling as essential for the homeostatic regulation of voltage-dependent calcium influx into cardiomyocytes. This mechanism provides the basis for a dynamic adjustment of the channel's surface availability and thus, of heart's contraction.

## Highlights

• Ca_V_1.2 channels dwell only about 10 min in the plasma membrane of atrial cells
• Ca_V_1.2 recycles via Rab11a endocytic recycling compartment along actin filaments
• Recycling of Ca_V_1.2 is essential for maintaining stable L-type current amplitudes
• Surface Ca_V_1.2 homeostasis is independent of tubulin-based transport over 20 hr

## Graphical Abstract

**Figure:**
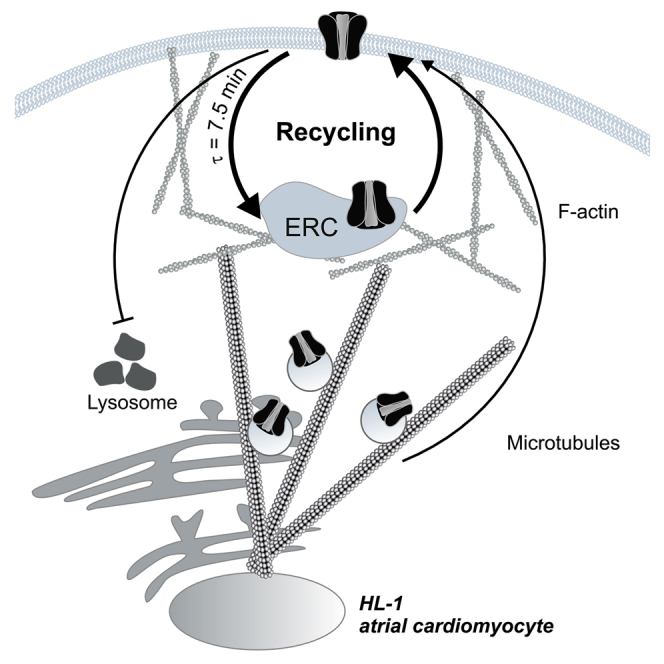

## Introduction

Depolarization of cardiomyocytes opens Ca_V_1.2 L-type voltage-activated calcium channels, allowing the influx of calcium ions, which in turn triggers calcium release from the sarcoplasmic reticulum, permitting effective myofilament contraction (Bodi et al., 2005). The Ca_V_1.2 core complex is composed of the Ca_V_1.2 α_1_ ion-conducting subunit, plus the accessory α_2_δ- and β-subunits that regulate the conduction properties and surface expression of the channel (Hofmann et al., 2014). The Ca_V_1.2 α_1_ and Ca_V_β_2_ subunits are essential for cardiac function. Mice bearing homozygous deletions of either gene die at embryonic stages (Larsen et al., 2002, Link et al., 2009, Rusconi et al., 2016, Seisenberger et al., 2000, Weissgerber et al., 2006). In humans, aberrant calcium permeation through Ca_V_1.2 channels is associated with several pathological cardiac conditions (Basheer and Shaw, 2016, Dick et al., 2016, Hofmann et al., 2014, Hong et al., 2012, Shaw and Colecraft, 2013, Splawski et al., 2004, Splawski et al., 2005). The function and number of channels at the plasma membrane regulates the amount of calcium entering into the cardiomyocytes. The cell surface abundance of Ca_V_1.2 is determined by the balance between the anterograde traffic that inserts channels into the plasma membrane via the secretory and the recycling pathways and the retrograde traffic that removes channels from the cell surface by endocytosis. The post-endocytic fate towards recycling or degradation can critically affect channel availability, but the extent to which these different trafficking events contribute to calcium current control is poorly understood.

Defective trafficking of the cardiac Ca_V_1.2 channel protein has been linked to disorders including atrial fibrillation, heart failure, and Brugada syndrome (Antzelevitch et al., 2007, Basheer and Shaw, 2016, Schotten et al., 2003, Simms and Zamponi, 2012, Xiao and Shaw, 2015). Thus, elucidation of the pathway by which Ca_V_1.2 traffics to and from the plasma membrane is very relevant to understanding heart function and dysfunction.

**Figure 1:**
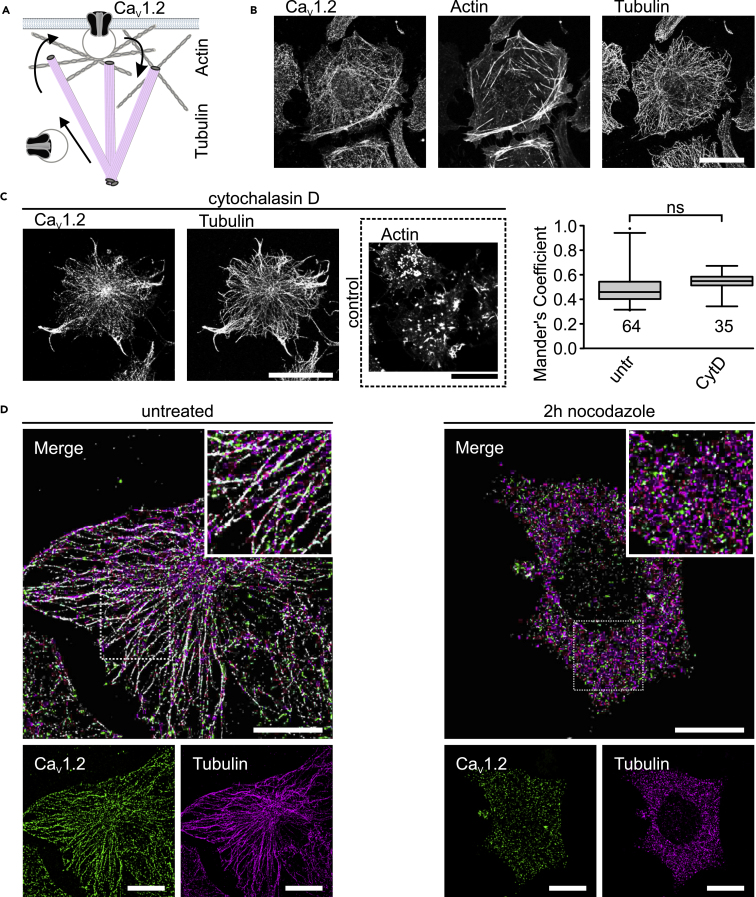
Ca_V_1.2 Distributes along Microtubules Extending from the Perinuclear Region to the Cell Periphery Independent of an Intact Actin Cytoskeleton (A) Schema of the intracellular anterograde and retrograde trafficking of the calcium channel. The channel is packed into vesicles that walks along tubulin and actin filaments to be delivered to the plasma membrane from where it can be removed by endocytosis during retrograde traffic. (B) Laser scanning confocal images of representative HL-1 cells fluorescently stained for Ca_V_1.2, tubulin and actin, using antibody coupled to ATTO 488, antibody coupled to Cy3 and phalloidin coupled to Alexa Fluor 647, respectively. Scale bar: 25 μm. (C) Laser scanning confocal images of HL-1 cells immunostained for Ca_V_1.2 and tubulin in the presence of 10 μM of the actin filament disrupter cytochalasin D and box plot summarizing the colocalization analysis between Ca_V_1.2 and tubulin according to Manders overlap coefficient in untreated and cytochalasin D treated HL-1 cells. The right panel shows a control cell exposed to the same cytochalasin D treatment and stained for actin using phalloidin-647. Scale bar: 25 μm. Numbers below each box correspond to the number of cells analyzed (*n* size). Each box represents the interquartile range (25^th^ and 75^th^ quartile) with the median indicated by the line. Whiskers above and below indicate the 95% confidence interval. Outliers are shown by dots. Statistical significance (one-way ANOVA, p ≤ 0.001). (D) Single-molecule localization microscopy images from representative HL-1 cells immunostained for Ca_V_1.2 (green) and tubulin (magenta) under normal conditions (left panel) and after 2 hr exposure to the microtubule disrupter nocodazole (right panel). The merged images are shown with an enlarged view of the marked square regions. Overlapping pixels appear in white. Single-molecule localizations were extracted from the data using SNSMIL (Tang et al., 2015). Scale bar: 10 μm for the bottom panels and 1 μm for the merge figure.

As for all membrane proteins, ion channels are incorporated into vesicles and directionally transported by motor proteins along two main cytoskeletal tracks, actin and tubulin filaments (Figure 1A). Classically, the microtubule network is considered the major pathway sustaining long-range intracellular vesicular transport, whereas actin filaments are involved in short-range transport processes at the cell cortex. However, long-range vesicle transport mediated by the actin cytoskeleton has been reported (Schuh, 2011). In cardiomyocytes, Ca_V_1.2 appears to be transported along microtubule tracks from early secretory compartments to the cell periphery. Here, the scaffolding protein BIN 1 is responsible for delivering the channel to the T-tubules (Hong et al., 2010). In addition, it has been demonstrated that actin filaments play a role in promoting cell surface insertion of Ca_V_1.2 channels, in atrial-derived HL-1 cells, via their direct association with the β-subunit (Stölting et al., 2015).

Much less is known about the endocytic route of Ca_V_1.2, although increasing evidence shows that internalization and degradation contribute to regulation of the Ca_V_1.2 cell surface expression (Best et al., 2011, Catalucci et al., 2009, Felix and Weiss, 2017, Green et al., 2007). Endocytosis and recycling are relatively fast (with half-time values around a couple of minutes [Maxfield and McGraw, 2004]), dynamic, and spatially confined trafficking events that may reversibly switch on and off the channel's cell surface availability, but the relevance of the endocytic pathway in modulating Ca_V_1.2 cell surface density in cardiac cells has not yet been established.

We investigated the trafficking of Ca_V_1.2 channels in HL-1 atrial cells. Our findings demonstrate that post-endocytic sorting is essential for governing Ca_V_1.2 surface availability, challenging the notion that microtubule-mediated transport is the rate-limiting step for maintaining stable Ca_V_1.2 currents (Hong et al., 2010). Paradoxically, we found that the channel turnover at the plasma membrane is relatively fast, with a time constant of internalization of about 7.5 min. We show that the loss of cell surface channels due to dynamic endocytosis is balanced by reinsertion of recycled channels, rather than of *de novo* synthesized protein, via a pathway mediated by Rab11a. This pathway is dependent on an intact actin cytoskeleton.

Our results may help to develop new strategies for treating Ca_V_1.2-associated channelopathies aimed at adjusting the number of expressed channels.

## Results

### Endogenous Ca_V_1.2 Localizes Along Radially Distributed Microtubules and Peripheral Actin Filaments in HL-1 Cells

We used three-color laser scanning confocal fluorescence microscopy to visualize the distribution of Ca_V_1.2 channels with respect to the actin- and tubulin-based cytoskeleton in HL-1 cells (Figure 1B). Immunostained Ca_V_1.2 forms distinct thread-like structures broadly distributed throughout the cell, extending from the perinuclear region to the cell cortex, with prominent accumulation at the cell periphery (Figure 1B, left panel). At periphery, immunostained Ca_V_1.2 appears to colocalize with phalloidin-stained actin filaments (Figure 1B, middle panel) probably reflecting the association of the channel complex and F-actin via the β-subunit, as previously reported in HL-1 cells (Stölting et al., 2015).

Ca_V_1.2 thread-like structures at the cell interior closely resemble the distribution of the microtubule network (Figure 1B, right panel). Quantitative analysis of the degree of colocalization between fluorescently labeled Ca_V_1.2 and microtubules from the confocal laser-scanning images, using Manders' overlap coefficient (MOC) (Bolte and Cordelieres, 2006), resulted in a moderate correlation value (0.49 ± 0.02, Figure 1C). This MOC value is not altered after treating the cells with 10 μM cytochalasin D, which effectively disrupts actin filaments (Figure 1C). This suggests that the delivery of Ca_V_1.2 to microtubule tracks does not require an intact actin-based cytoskeleton.

To study the spatial correlation between Ca_V_1.2 and tubulin at nanoscale resolution, we used single-molecule localization microscopy (SMLM) on immunofluorescently stained HL-1 cells, as previously described (Stölting et al., 2015). SMLM images from HL-1 cells immunolabeled for Ca_V_1.2 and tubulin show that Ca_V_1.2 distributes along microtubules over several micrometers, from the microtubule-organizing center adjacent to the nucleus to the cell periphery (Figure 1D). Pharmacological disruption of the microtubule network using nocodazole resulted in a spotty distribution of Ca_V_1.2 and tubulin and a loss of the spatial correlation between the channel protein and unpolymerized tubulin subunits (Figure 1D).

Altogether, these results indicate that transport of Ca_V_1.2 from the early secretory compartments towards the cell periphery takes place along microtubules, and independently of actin filaments. Moreover, they show that a major fraction of the intracellular Ca_V_1.2 pool associates with cytoskeletal tracks, indicating a role for trafficking processes in regulating channel availability.

### Pharmacological Disruption of the Microtubule Network Preserves Endogenous L-type Current Expression in HL-1 Cells

**Figure 2:**
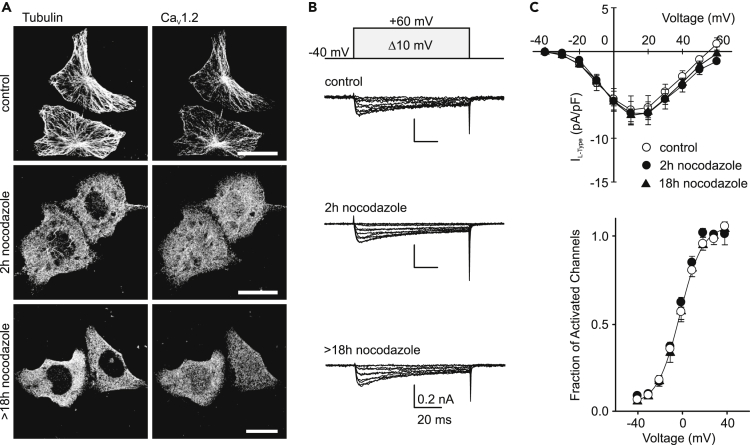
Long-term Stable Expression of L-type Currents in HL-1 Cells Is Independent of Microtubule-Based Transport (A) Laser scanning confocal images of control HL-1 cells immunostained for tubulin and Ca_V_1.2 (top panels) or cells following incubation with 33 μM of nocodazole for 2 hr (middle panels) or 18–22 hr (bottom panels). Scale bar: 25 μm. (B) Representative L-type current traces from cells under the three conditions shown in *A* elicited by voltage steps from a holding potential of −40 mV to +60 mV in 10 mV increments (protocol pulse indicated above). (C) Average current density to voltage (I-V) curves (upper panel) and fraction of activated channels versus voltage plot (activation curves, lower panel) for L-type currents (I_L-type_) from different cells as shown in *B* (n = 5 for control cells and n = 6 and 5, for cells exposed to nocodazole for 2 hr and >18 hr, respectively). The continuous line represents the fit to a Boltzmann function for the control dataset.

Next, we examined the extent to which microtubule-dependent transport participates in the modulation of Ca_V_1.2 cell surface expression using the whole-cell patch-clamp technique. L-type currents were recorded from control HL-1 cells and from cells treated with nocodazole for either 2 or 18 hr (Figure 2). The efficacy of nocodazole treatment over the different durations was evaluated by double immunofluorescence labeling of tubulin and Ca_V_1.2 (Figure 2A). Confocal images show that after 2 hr of exposure to nocodazole the microtubule network is almost fully disrupted, and following 18 hr of treatment, virtually no filaments are visible. In addition, since prolonged nocodazole treatment may lead to Golgi scattering and thus artificial delivery of the channel from endoplasmic reticulum (ER)-Golgi to the plasma membrane (Cole et al., 1996), we also investigated the architecture of the Golgi compartment in cells exposed to the same nocodazole regime as for the electrophysiological recordings. Cells immunostained with a trans-Golgi antibody showed the typical Golgi ribbon structure localized in the perinuclear region (Figure S1). After 2 hr exposure to nocodazole, the cells showed a relatively less compacted juxtanuclear Golgi structure, and after 18 hr of treatment significantly more scattered fragments were observed throughout the cell (Figure S1). Despite the progressive increase in Golgi fragmentation over time of exposure, the L-type current density amplitudes and voltage dependence of activation were preserved (Figures 2B and 2C). Thus, Golgi scattering does not correlate with an increase in L-type currents. Both groups of treated HL-1 cells (for 2 and >18 hr) showed identical current amplitudes and voltage dependence as control cells demonstrating that nocodazole treatment has no effect on both the biophysical properties of the Ca_V_1.2 channel and the number of functional channels assembled at the plasma membrane (Figures 2B and 2C). This lack of effect of nocodazole indicates that either the lifetime of the channel at the plasma membrane is relatively long (>20 hr) or, alternatively, a mechanism independent of microtubule-based transport is responsible for maintaining steady-state levels of cell surface Ca_V_1.2.

### Ca_V_1.2 Channels Are Internalized with a Time Constant of a Few Minutes in HL-1 Cells

**Figure 3:**
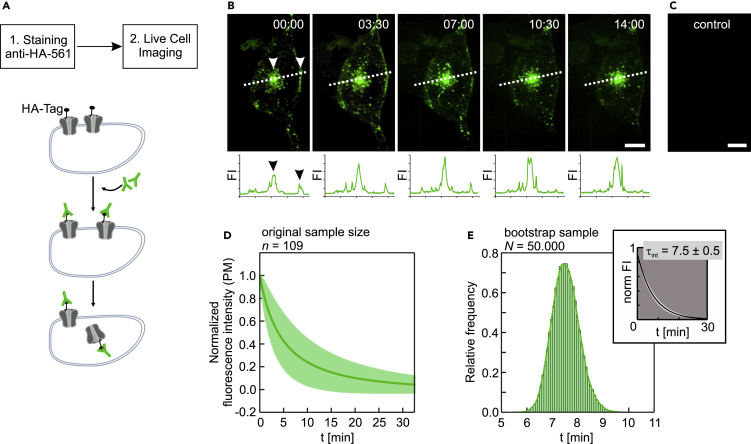
Internalization of Ca_V_1.2 Channels in HL-1 Cells Occurs within a Few Minutes (A) Experimental design to follow the time course of Ca_V_1.2 internalization. HL-1 cells expressing extracellularly HA-epitope tagged Ca_V_1.2 are incubated with fluorescently labeled anti-HA-antibody at room temperature, washed, and immediately transferred to the cell microscope stage top incubator set at 37°C for live cell imaging using the spinning disk confocal microscopy. (B) Representative time-lapse image sequence of the course of internalization of fluorescently labeled Ca_V_1.2 in HL-1 cells. The time after completion of the labeling reaction at which each frame was acquired is indicated. The corresponding fluorescence intensity plots along the dotted line drawn by eye across the middle of the cell are shown in the lower panels. The white arrowheads in the image shown at t = 0 indicate HA-labeled channels accumulated at the cell center and at the plasma membrane and correspond to the black arrowheads in the fluorescence intensity profile shown below. Over time the fluorescence intensity in the plasma membrane decreases, whereas the fluorescence intensity in the inner part of the cell increases. Scale bar: 10 μm. (C) Non-transfected control HL-1 cells show no HA-mediated signal after incubation with the fluorescently labeled anti-HA tag antibody. Scale bar: 10 μm. (D) Mean time course of Ca_V_1.2 internalization (continuous dark green line) and SD (light green shaded area) of all the individual normalized exponential fits to the fluorescence decay of the different regions of interest at the plasma membrane (PM) from different cells analyzed (original sample size *n* = 109). (E) Distribution of the time constants obtained from the bootstrap samples. The mean time constant ± SD of internalization (*τ*_*int*_) and confidence interval (CI) extracted from the bootstrap distribution is 7.5 ± 0.5 min with 95% CI from 6.55 to 8.65 min. The insert shows the mean time course of Ca_V_1.2 internalization (black line) and SD (white shaded area) of the individual exponential fits to all the bootstrap samples (bootstrap sample *N* = 50,000).

We assessed the average residence time of Ca_V_1.2 channels at the plasma membrane of HL-1 cells. Cells were transfected with a cDNA construct encoding Ca_V_1.2 bearing an extracellular hemagglutinin (HA) epitope (Ca_V_1.2-HA). To selectively label channels at the cell surface, Ca_V_1.2-HA-expressing cells were briefly exposed to a fluorophore-conjugated anti-HA antibody, and the time course of subsequent channel internalization was monitored at 37°C using spinning disk confocal microscopy, as shown in Figures 3A–B. The anti-HA-mediated fluorescence signal was found at the plasma membrane and throughout the cytoplasm in small punctuate structures and was concentrated at the cell interior (Figure 3B). The fluorescence intensity plots across a dotted line within the cell, obtained from the image sequence, show that the membrane staining decreases relatively rapidly over time, whereas the intracellular signal steadily increases (Figure 3B, lower panel). Neither membrane staining nor intracellular retention of the antibody was observed in untransfected cells (Figure 3C).

To estimate the lifetime of the channel at the cell surface, the fluorescence intensity in channel clusters at the plasma membrane from different regions of interest, and in several different cells, were plotted as a function of time and fitted individually to a single exponential, as described in the Transparent Methods section. The mean normalized fitted exponential and the SD for all the analyzed regions of interest is shown in Figure 3D. Using the bootstrap method (Efron and Tibshirani, 1993), the mean time constant of internalization (*τ*_*int*_) ± SD for Ca_V_1.2 was calculated from the bootstrap sample distribution, obtaining a value for *τ*_*int*_ of 7.5 ± 0.5 min (Figure 3E). To compare the total protein expression levels of Ca_V_1.2 in transfected and untransfected cells, we calculated the fluorescence intensity of immunolabeled Ca_V_1.2. We found that Ca_V_1.2 expression levels are comparable in both cases, and thus, we assume that the calculated time constant holds for endogenous expressed channels (Figure S2).

Since we observed that cell surface levels of Ca_V_1.2 are preserved for over 20 hr after disruption of the microtubule network, the relatively rapid Ca_V_1.2 internalization rate predicts the existence of dynamic trafficking events that must replace the internalized channels by newly inserted ones, independent of a competent microtubule-based cytoskeleton.

We used a dual-pulse staining protocol to estimate the rate at which new Ca_V_1.2 channels are inserted into the plasma membrane (Figure S3). The first pulse saturates all Ca_V_1.2 present at the cell surface, whereas the second pulse, 20 min later, labels only channels that have been inserted during the time elapsed between the two pulses, i.e., newly inserted channels. We observed a robust staining of Ca_V_1.2 channels during the second pulse, indicating that a dynamic insertion of new channels into the plasma membrane takes place during a time window of about 20 min. As predicted from the lack of effect of nocodazole treatment on the cell surface expression of Ca_V_1.2 (Figure 2), the newly inserted channels originate from the recycling rather than from the microtubule-based secretory pathway, as is shown below. Since we do not observe the fluorescence signal returning to the plasma membrane, we assume that the fluorescently labeled antibody dissociates from the channel during the post-endocytic recycling itinerary.

Altogether, the above findings show that the cell surface Ca_V_1.2 turnover is very dynamic, that it occurs on a relatively short timescale of tens of minutes, and that internalized Ca_V_1.2 channels are effectively replaced by newly delivered Ca_V_1.2 protein.

### Endogenous Ca_V_1.2 Is Internalized via Clathrin-Mediated Endocytosis and Diverted to Rab11-Positive Recycling Endosomes but Not to Lysosomes

**Figure 4:**
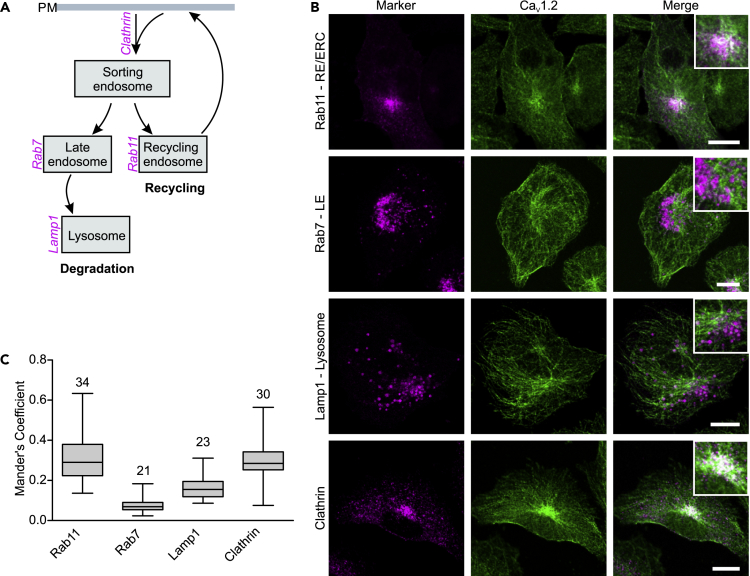
Endogenous Ca_V_1.2 Colocalizes with Clathrin-Endocytic Vesicles and Rab11a-Recycling Endosomes but Not with Lysosomes (A) Illustration showing a general endocytic pathway with the endosomal markers used in this study. HL-1 cells were immunostained for Ca_V_1.2, clathrin, Rab7 (late endosomes, LE), and Rab11a (recycling endosomes and endocytic recycling compartment, RE/ERC). Lysosomes were labeled using a cDNA encoding for LAMP1 fused to monomeric red fluorescent protein (mRFP). PM, plasma membrane. (B) Laser scanning confocal images of HL-1 cells fluorescently labeled for endogenous Ca_V_1.2 (green) and the indicated endocytic pathway marker (magenta). The corresponding merge images with an enlarged view are shown at the right. Overlap pixels appear in white. Scale bar: 10 μm. (C) Box plot summarizing the colocalization analysis between Ca_V_1.2 and the indicated markers according to Manders' overlap coefficient in HL-1 cells. Numbers above each box represent the number of cells analyzed (*n* size). Each box represents the interquartile range (25^th^ and 75^th^ quartile) with the median indicated by a line. Whiskers above and below indicate the 95% confidence interval. Ca_V_1.2 colocalizes with clathrin and Rab11-positive endosomes but not with late endosomes (Rab7-positive) or lysosomes (Lamp1).

We next investigated the endosomal transport of endogenously expressed Ca_V_1.2 in HL-1 cells by quantifying its degree of colocalization with different endosomal markers along the endocytic pathway using the MOC (Figure 4).

Internalization of receptors and ion channels may occur via clathrin-mediated and clathrin-independent endocytosis. After internalization, the protein is transferred to sorting endosomes and, from there, either recycled back to the plasma membrane by the so-called fast or slow recycling or transported via late endosomes to lysosomes for degradation (McMahon and Boucrot, 2011). During slow recycling, mediated by Rab11 GTPases, cargo proteins are first transferred to the endocytic recycling compartment (ERC) before being recycled back to the plasma membrane with a half-life of around 12 min (Hao and Maxfield, 2000). Fast recycling, mediated by Rab4 GTPases, return cargo to the cell surface directly from the sorting endosomes approximately 10 times faster than slow recycling (Hao and Maxfield, 2000, Maxfield and McGraw, 2004).

We analyzed the spatial correlation of Ca_V_1.2 with clathrin, and also with Rab11a, a marker for recycling endosomes (RE) and the perinuclear ERC, and also of Ca_V_1.2 with the degradation pathway markers, Rab7 (for late endosomes) and LAMP1 (for lysosomes) (Figure 4A). The laser scanning confocal images show that Ca_V_1.2 distribution overlaps with that of clathrin and Rab11a, but not with that of Rab7 and Lamp1 (Figures 4B and 4C). The MOC values obtained show a modest, but significantly higher degree of colocalization between Ca_V_1.2 and either clathrin or Rab11a than is observed between Ca_V_1.2 and either Rab7-positive late endosomes or lysosomes (Figure 4C).

These results demonstrate that endogenous Ca_V_1.2 is partly internalized via clathrin-mediated endocytosis and is mainly translocated via Rab11a to the ER/ERC for recycling, thus escaping lysosomal degradation. Recycling of Ca_V_1.2 appears to be the main itinerary during the endocytic trafficking of Ca_V_1.2.

### Translocation of Ca_V_1.2 to the Rab11a Perinuclear Endocytic Recycling Compartment Depends on an Intact Actin Cytoskeleton but Not on the Microtubule Network

**Figure 5:**
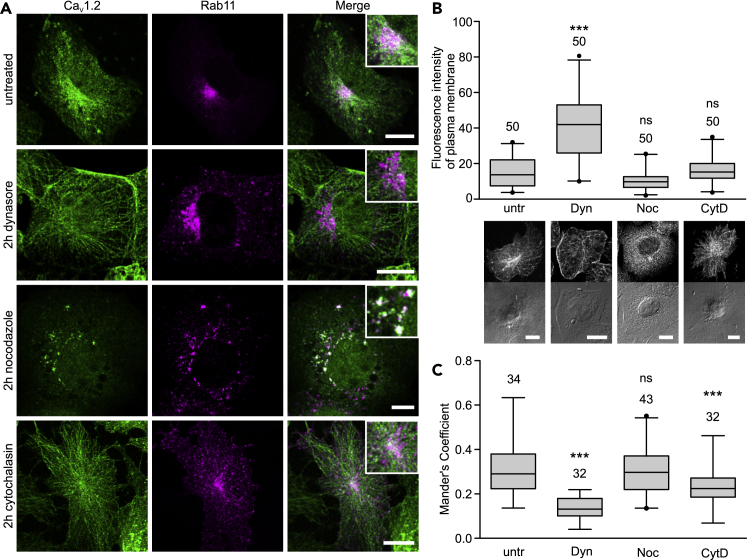
Targeting of Endogenous Ca_V_1.2 to Rab11a-recycling Compartment in HL-1 Cells Depends on an Intact Actin Cytoskeleton but Not on Microtubules (A) Laser scanning confocal images of HL-1 expressing Rab11a-mRFP (magenta) and immunostained for Ca_V_1.2 (green) after 2 hr exposure to the indicated pharmacological agents: dynasore to inhibit clathrin-mediated endocytosis, nocodazole and cytochalasin D to disrupt the microtubule- and actin-based cytoskeleton, respectively. The overlapping pixels appear in white in the merged images shown at the right. Inset shows an enlarged view of the corresponding merged image. Scale bars: 10 μm. (B) Box plot of the normalized fluorescence intensity at the plasma membrane from cells treated as described in panel (A). For better visualization of the cell membrane, the fluorescence and transmission images of representative cells are shown below. Scale bars: 10 μm. untr, Untreated cells; Dyn, dynasore; Noc, nocodazole; CytD, cytochalasin D. Numbers above each box represent the *n* size. Each box represents the interquartile range (IQR) (25^th^ and 75^th^ quartile) with the median indicated by a line. Whiskers above and below indicate the 95% confidence interval. Outliers are shown by dots. Statistical significance (one-way ANOVA, p ≤ 0.001). (C) Box plot of the colocalization analysis between Ca_V_1.2 and Rab11a, according to Manders' overlap coefficient evaluated after 2 hr of exposure to the indicated drug. Numbers above each box represent the *n* size. Each box represents the IQR (25^th^ and 75^th^ quartile) with the median indicated by a line. Whiskers above and below indicate the 95% confidence interval. Outliers are shown by dots. Statistical significance (one-way ANOVA, p ≤ 0.001).

We anticipate that recycling mediated by the actin cytoskeleton is responsible for maintaining stable surface expression of Ca_V_1.2 during inhibition of microtubule-based transport (Figure 2). To investigate the dependence of Rab11a-mediated Ca_V_1.2 recycling on cytoskeletal components, we analyzed the effect of nocodazole and cytochalasin D on the degree of Ca_V_1.2/Rab11a colocalization (Figure 5). Since during clathrin-mediated endocytosis, dynamin is used for the scission of the endocytic vesicle from the plasma membrane (Maxfield and McGraw, 2004), we used the dynamin-dependent endocytosis inhibitor, dynasore, as control. Inhibition of endocytosis decreases the incoming endosomal traffic, and thus it is expected to increase the cell surface expression of Ca_V_1.2 and to reduce its entry to the ERC.

HL-1 cells were transfected with a construct encoding a fluorescently labeled Rab11a (Rab11-mRFP), exposed to the corresponding inhibitor for 2 hours, and then immediately fixed and immunostained for Ca_V_1.2. The suitability of the Rab11-mRFP construct for selectively labeling the ERC was assessed by immunodetection of the fluorescently labeled Rab11a with the anti-Rab11a antibody used in Figure 4. The fluorescence signals overlapped almost completely in the perinuclear region (Figure S4). Cells treated with dynasore show a significant enrichment of the Ca_V_1.2 fluorescence signal at the plasma membrane and a decreased degree of colocalization between Ca_V_1.2 and Rab11a, compared with that in non-treated cells (Figure 5). In time-lapse experiments, heterologously expressed HA-tagged Ca_V_1.2 exhibit longer dwelling times at the plasma membrane of dynasore-treated cells than of untreated cells (Figure S5).

In contrast, exposure of HL-1 cells to nocodazole has no effect on the surface density of the channel, as demonstrated by both electrophysiology (Figure 2) and fluorescence intensity at the cell membrane (Figure 5B), or on its overlap with the Rab11a ERC (Figure 5C). Using double staining for Golgi and Rab11a-ERC, we excluded the possibility that fragmentation of these compartments induced by nocodazole could lead to random overlap between the corresponding scattered fragments (Figure S6).

Depolymerization of actin filaments by cytochalasin D also impaired the delivery of Ca_V_1.2 into the ERC, as manifested by the decreased MOC value for Ca_V_1.2 and Rab11a (Figure 5C). However, the surface expression of the channel, as shown electrophysiologically here (Figure 5B) and in a previous report (Stölting et al., 2015), was not altered, in contrast to the results using dynasore.

These results show that Rab11a-mediated translocation of Ca_V_1.2 to the ERC depends on an intact actin- but not microtubule-based cytoskeleton. They also suggest the existence of diverse cellular strategies for maintaining stable levels of Ca_V_1.2 at the cell surface by compensating for trafficking dysfunction. However, we found that only impairment of endocytosis results in an excess of channels at the plasma membrane (Figure 5) suggesting that this process is central for the homeostatic regulation of cell surface Ca_V_1.2.

### Disruption of the Actin Cytoskeleton Re-Routes Internalized Ca_V_1.2 from Recycling to Lysosomal Degradation

**Figure 6:**
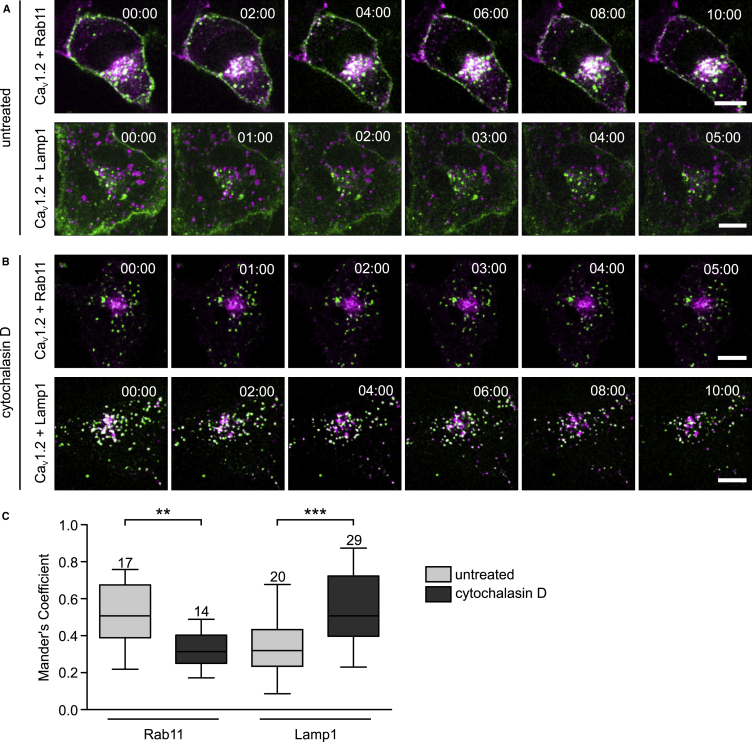
Disruption of the Actin Cytoskeleton Re-routes Ca_V_1.2 to Lysosomes in HL-1 Cells (A) Time-lapse sequences of the sorting of internalized Ca_V_1.2 in HL-1 cells expressing either fluorescently labeled Rab11a or LAMP1 for staining RE/ERC and lysosomes, respectively. Ca_V_1.2-HA channels at the cell surface were labeled as in Figure 3. The HA-mediated fluorescence signal of the cell surface Ca_V_1.2 channels is shown in green, and the signal for Rab11a-mRFP and LAMP1-mRFP, in magenta. Only the merged images are shown with the overlapping pixels in white. Scale bar: 10 μm. (B) Time-lapse sequences of the sorting of internalized Ca_V_1.2 in HL-1 cells as in *A*, but after pharmacological disruption of the actin cytoskeleton using cytochalasin D. Post-endocytic accumulation of Ca_V_1.2 in the RE/ERC is severely impaired in the absence of an intact actin cytoskeleton. Scale bar: 10 μm. (C) Box plot of the colocalization analysis according to Manders' overlap coefficient between Ca_V_1.2 with either Rab11a or LAMP1 in HL-1 cells in the presence (dark gray boxes) and absence (light gray boxes) of cytochalasin D. MOC was calculated from the images acquired at t = 0. Numbers above each box represent the *n* size. Each box represents the interquartile range (25^th^ and 75^th^ quartile) with the median indicated by a line. Whiskers above and below indicate the 95% confidence interval. Statistical significance (one-way ANOVA, ***p ≤ 0.001; **p ≤ 0.01).

To test whether the observed colocalization of Ca_V_1.2 with Rab11a reflected sorting of newly internalized, rather than *de novo* synthesized, channels into the perinuclear ERC, we monitored the post-endocytic fate of Ca_V_1.2 in living cells. HL-1 cells cotransfected with Ca_V_1.2-HA and either fluorescently labeled Rab11a or Lamp1 were subjected to cell surface labeling reaction and imaging using spinning disk microscopy, as described in Figure 3. Time-lapse sequences of the merged images for non-treated cells and cells treated with cytochalasin D are shown in Figure 6. In non-treated HL-1 cells, internalized Ca_V_1.2 channels are mostly translocated, via Rab11a-positive endosomes, to the ERC where they accumulate over time (Figure 6A, top panel) and delivery of channels to lysosomes (LAMP1) is only marginal (Figure 6A, bottom panel). However, the post-endocytic fate of the channel protein changes dramatically upon disruption of actin filaments with cytochalasin D (Figure 6B). Under such conditions, internalized Ca_V_1.2 is virtually excluded from the Rab11a-RE/ERC, whereas most of the channels are translocated to lysosomes (Figure 6B).

We estimated the MOC values for Ca_V_1.2 with either Rab11a or Lamp1 in living cells from the first images of each time-lapse sequence (t = 0). The MOC values show that the degree of overlap changes significantly in the presence of cytochalasin D (Figure 6C). Disruption of the actin cytoskeleton resulted in a decrease of the mean MOC value for Ca_V_1.2 and Rab11a from 0.52 to 0.32, but produced an increase in overlap between Ca_V_1.2 and Lamp1 from 0.32 to 0.55.

These results demonstrate that disruption of the actin cytoskeleton re-routes endocytosed Ca_V_1.2 from recycling to lysosomal degradation. The post-endocytic sorting of Ca_V_1.2 to the recycling compartment therefore relies on a competent actin cytoskeleton.

## Discussion

Normal cardiomyocyte excitability and contractility rely on controlled calcium entry via Ca_V_1.2 channels. Endocytosis decreases the availability of channels at the plasma membrane, but its contribution to Ca_V_1.2 regulation is unclear. To maintain steady-state levels of Ca_V_1.2 at the plasma membrane, the loss of channels due to endocytosis might be compensated by the insertion of new channels from the secretory and/or recycling pathways. Our understanding of Ca_V_1.2 trafficking pathways in native systems has been limited, partly due to the restricted capability to visualize and dissect channels originating from the different trafficking routes.

Here, using spinning disk microscopy and fluorescence-specific labeling of cell surface Ca_V_1.2 and the endosomal-lysosomal compartments, we follow the post-endocytic fate of the channel in HL-1 atrial-derived cells. We demonstrated that endocytic recycling mediated by Rab11a is a major trafficking route regulating stable L-type current expression. Ca_V_1.2 is efficiently internalized via the clathrin-dynamin-mediated pathway and mostly translocated to the recycling compartment via Rab11a, while escaping lysosomal degradation. In ventricular myocardium, Rab11b, another member of the Rab11 family, drives the degradation of cell surface Ca_V_1.2 channels (Best et al., 2011). Therefore, either different members of the Rab11 family may differently regulate the endocytic fate of the channel or, alternatively, Rab11-mediated endosomal transport of Ca_V_1.2 may be tissue-specific.

Perturbation of the microtubule network for at least 20 hr does not alter channel abundance at the surface of HL-1 cells (Figure 2). This suggests that Ca_V_1.2 can cycle between the plasma membrane and the ERC for at least this period of time without secretory traffic input. In HEK cells the half-life of total cellular Ca_V_1.2 was calculated to be 3.5 hr (Chien et al., 1995). This rather short half-life possibly reflects the lack of regulatory mechanisms that control channel trafficking, stability, and degradation in heterologous expression systems.

Depolymerization of actin filaments by cytochalasin D impairs the translocation of endocytosed Ca_V_1.2 to the RE/ERC and diverts the channel to lysosomes (Figure 6), indicating that the entry to the ERC is actin dependent, whereas translocation to lysosomes is not. The internalization of channels in the absence of F-actin suggests that the delivery to the first station along the endocytic pathway, the sorting endosome, is independent of an intact actin cytoskeleton. These results are consistent with the finding that *in vitro* reconstitution of the transferrin receptor transport to recycling endosomes is inhibited by cytochalasin D (Bartz et al., 2003) but its delivery to early/sorting endosomes persists during disruption of the actin cytoskeleton by latrunculin B (Ohashi et al., 2011). We assume that the transport of Ca_V_1.2 from sorting endosomes to the ERC and from the ERC back to the plasma membrane is mediated by the actin-based motor proteins, myosin V and VI, as reported for other ion channels in cardiac myocytes (Collaco et al., 2010, Lapierre and Goldenring, 2005, Lapierre et al., 2001, Millman et al., 2008, Schumacher-Bass et al., 2014, Swiatecka-Urban et al., 2004, Swiatecka-Urban et al., 2007).

We estimated an unprecedented short time constant of internalization of 7.5 ± 0.5 min for Ca_V_1.2 in HL-1 cells. To our knowledge, this is the first reported *τ*_*int*_ for Ca_V_1.2 in cardiomyocytes. Comparably short times have been obtained only for the K_V_1.5 voltage-gated potassium channel in HL-1 cells, wherein efficient recycling mediated by Rab11 and Rab4 proved to be relevant for maintaining steady-state levels of the cell surface channels (McEwen et al., 2007). As the cell surface density of Ca_V_1.2 channels remains unaltered in cells treated with cytochalasin D (Stölting et al., 2015 and Figure 5), we envisage that Rab4-mediated fast recycling maintains channel homeostasis under these conditions. Figure S2 shows that a fraction of newly inserted channels overlaps with preassembled channels stained during the first pulse at the cell periphery. Thus, fast recycling may provide a confined trafficking circuit to regulate local Ca_V_1.2-mediated signaling.

The question arises why is Ca_V_1.2 exposed to such a high turnover rate at the cell surface of heart cells? We propose that the dynamic endocytic recycling of Ca_V_1.2 provides not only a quantitative regulation of the amount of channels at the plasma membrane but also a qualitative control needed to ensure the expression of functional Ca_V_1.2 channel complexes at the plasma membrane. Ca_V_1.2 is subjected to extensive regulation by a variety of mechanisms that are vital for cardiac function under normal and stress conditions. These include different types of post-translational modifications and dynamic associations with different modulatory proteins (Oz et al., 2017, Simms et al., 2015). Channel-ligand interactions and post-translational modifications may alter the protein-protein interaction environment of the channel and introduce local unfolding, prompting the channel to undergo quality control.

Among post-translational modifications, phosphorylation of Ca_V_1.2 via β1-adrenergic receptor activation is a well-established event that facilitates calcium current during the fight-or-flight response (Huang and Zamponi, 2017, Hulme et al., 2006, Oz et al., 2017). Most recently, it has been shown that phosphorylation of Ca_V_1.2 at one specific site induces a conformational change to the channel and phosphorylation at a further site leads to the displacement of the β-adrenergic receptor (Cserne Szappanos et al., 2017, Patriarchi et al., 2016). Other phosphorylation events also cause alterations to the integrity of the Ca_V_1.2 macromolecular complex (Altier et al., 2012). Furthermore, phosphorylation of several ion channels, including connexin 43 and K_V_1.3, induces ubiquitination, followed by internalization and degradation, of these proteins (Martinez-Marmol et al., 2017, Smyth et al., 2014). Ubiquitination and deubiquitination of ion channels, including voltage-gated calcium channels, emerge as regulatory mechanisms controlling their proteosomal degradation after ER exit (Altier et al., 2011, Waithe et al., 2011) and their internalization rate and endosomal sorting (Eaton et al., 2010, Felix and Weiss, 2017, Garcia-Caballero et al., 2014, McCann et al., 2016). The K_ATP_ channel also undergoes rapid internalization and recycling back to the cell surface, and is diverted to lysosomal degradation upon activation of protein kinase C (PKC) (Manna et al., 2010). Conversely, a recent study reported that PKC phosphorylation of Ca_V_1.2 increases the expression of the channel at the plasma membrane of HL-1 cells (Keren Raifman et al., 2017). As already proposed for Cx43 (Smyth et al., 2014), specific timing and arrangement of phosphorylations of Ca_V_1.2 might determine the mode of ubiquitination and, in turn, the fate of the internalized protein.

**Figure 7:**
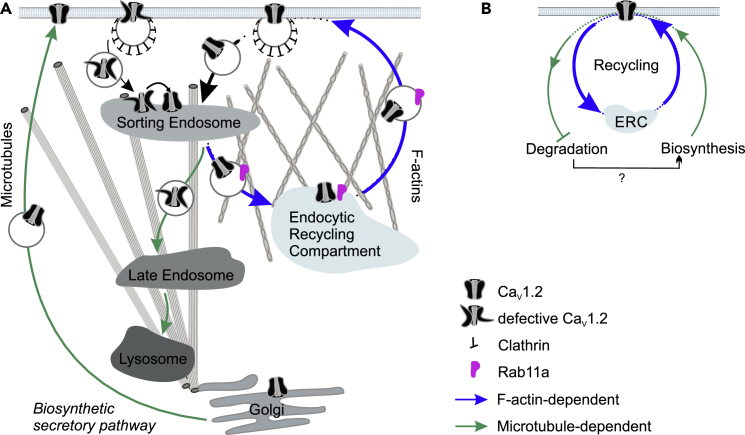
Model for Trafficking Pathway and Regulation of Cell Surface Expression of Ca_V_1.2 in HL-1 Cells (A) Ca_V_1.2 in HL-1 cells is internalized via clathrin-dynamin-dependent endocytosis and mainly recycled back to the plasma membrane via a Rab11a-mediated pathway. The delivery of the channel from sorting endosomes to the endocytic recycling compartment (ERC) and back to the plasma membrane is regulated by actin filaments (blue arrows). Traffic of defective channels from sorting endosomes to lysosomes, as well as of *de novo* channels from the biosynthetic secretory pathway, is mediated by microtubules (green arrows). (B) The secretory and endosomal transport of Ca_V_1.2 is depicted as interwoven trafficking pathways. The secretory traffic aids the refilling of the ERC pool upon removal of the channel from the recycling circuit by degradation, via a yet-to-be discovered crosstalk (black arrow).

We propose a model in which the post-endocytic trafficking of Ca_V_1.2 supports the quality control of the channel protein at the sorting endosome (Figure 7A). In this dedicated logistic center, the sorting fate of the Ca_V_1.2 toward one of two distinct routes, recycling or degradation, is decided according to the channel state and cellular demand. Corrupted channels are translocated to the degradation pathway via microtubule-based transport, whereas the remainder, including post-translationally modified ones, are repaired and, after quality control approval, are recycled back to the plasma membrane via a Rab11a-mediated process along actin filaments. The β-subunit associates directly with F-actin and stimulates the channel surface insertion (Stölting et al., 2015). This subunit may act as a tether to retain internalized channels and confine Ca_V_1.2-containing endosomes to the cortical actin while preventing the switch to microtubule tracks and diversion to lysosomes. The dynamic interaction of the β-subunit with Ca_V_1.2 fulfills a critical role in mediating quality control through endocytic recycling; association of the β-subunit with Ca_V_1.2 ensures normal channel function and survival at the cell surface, whereas its dissociation triggers endocytosis via the dynamin-dependent pathway (Gonzalez-Gutierrez et al., 2007, Hidalgo et al., 2006, Hidalgo and Neely, 2007, Miranda-Laferte et al., 2011). We hypothesize that during internalization Ca_V_1.2 and Ca_V_β traffic separately, whereas during forward transport either along the secretory or the recycling pathways, the two subunits associate with each other.

A key remaining question is what is the role of the secretory pathway in Ca_V_1.2 cell surface homeostasis. After all, a dynamic quality control at the plasma membrane requires active input of *de novo* channels. We found that a large fraction of Ca_V_1.2, presumably arising from the biosynthetic secretory pathway (Hong et al., 2010), co-distributes lengthwise with tubulin filaments extending from the perinuclear region to the cell periphery (Figure 1). This pool of intracellular channels may provide a reservoir for rapid secretory traffic to respond to physiological demand. We did not observe Ca_V_1.2 accumulation after disrupting the actin- and microtubule-based transport, suggesting a regulated rather than a constitutive channel biosynthesis. We envision that the pool of channels residing at the ERC is being constantly monitored and refilled by the secretory pathway through a yet-to-be-established feedback loop that matches degradation and protein translation rates (Figure 7B).

In summary, our findings reveal that the main sorting fate for endocytosed Ca_V_1.2 is recycling via the Rab11a-mediated pathway. Defects in Rab11-mediated recycling have been associated with several neurological diseases (Li and DiFiglia, 2012), but to date such aberrations have not been directly linked to cardiac disorders. A single point mutation in Ca_V_1.2 that alters the cell surface density of the channel has been found in a patient with Brugada syndrome (Antzelevitch et al., 2007). It will be interesting to investigate the recycling pathway of Ca_V_1.2 channels bearing this disease-causing mutation. The present results may help understanding Ca_V_1.2-trafficking-associated channelopathies and considering new therapeutic perspectives.

### Limitations of the Study

Adult cardiomyocytes are not suited for gene transfer of high-molecular-mass cDNA constructs as the one encoding for Ca_V_1.2 (Christensen et al., 2000, Christensen et al., 2003, Gizak et al., 2009, Louch et al., 2011) and they exhibit alterations in protein expression levels, peak L-type Ca^2+^ currents and channel localization, as well as cell morphology during the first 24 hr of culture (Banyasz et al., 2008, Leach et al., 2005, Louch et al., 2004, Mitcheson et al., 1996). This greatly restricts the use of this system for determining the internalization rate and fate of Ca_V_1.2 and impairs prolonged drug treatment of the cardiomyocytes (Figure 2). In contrast, HL-1 cells retain a rather embryonic phenotype but are well suited for studying trafficking of Ca_V_1.2.

Although, we cannot prove that our conclusions are valid for adult cardiomyocytes, we believe that the latter is subjected to even a tighter regulatory input to assure proper Ca_V_1.2-mediated calcium signals. Thus, a comparable dynamic recycling as described for HL-1 cells would be required for maintaining the quality control of the post-translated modified channels. Nevertheless, an additional trafficking route has been described in differentiated mouse cardiomyocytes that delivers Ca_V_1.2 to the T-tubules along microtubules via BIN1 protein (Hong et al., 2010). Likely, several trafficking routes assuring tight and local control of Ca_V_1.2 at the plasma membrane coexist in adult cardiomyocytes to support their increased demand for regulatory input.

## Methods

All methods can be found in the accompanying Transparent Methods supplemental file.

## Supplemental Information

Document S1. Transparent Methods and Figures S1–S6
