# Supplementary material for: Rapid Turnover of the Cardiac L-Type CaV1.2 Channel by Endocytic Recycling Regulates Its Cell Surface Availability
Source: iScience. 2018 Aug 16;7:1–15. doi: 10.1016/j.isci.2018.08.012 (PMC6135870; doi:10.1016/j.isci.2018.08.012)
Supplement: Document S1. Transparent Methods and Figures S1–S6 [file mmc1.pdf]

ISCI, Volume 7

Supplemental Information

Rapid Turnover of the Cardiac L-Type $Ca_V1.2$ Channel by Endocytic Recycling Regulates Its Cell Surface Availability

Rachel Conrad, Gabriel Stölting, Johnny Hendriks, Giovanna Ruello, Daniel Kortzak, Nadine Jordan, Thomas Gensch, and Patricia Hidalgo

Supplemental Figures

Figure S1

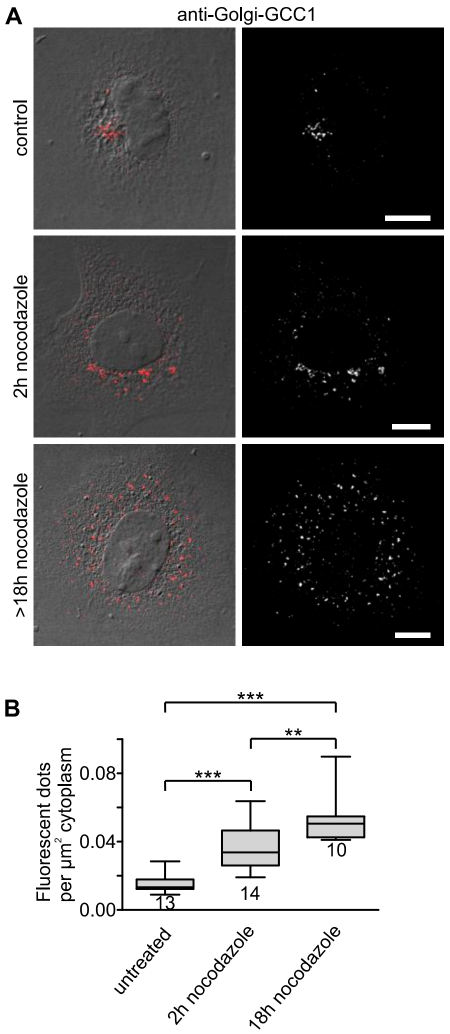

Figure S1. Prolonged nocodazole treatment induces Golgi scattering without changes in the peak amplitude of L-type currents, Related to Figure 2.

(A) Laser scanning confocal images of HL-1 cells stained with anti-Golgi-GCC1 antibody in untreated (control) cells and after incubation with nocodazole for either 2 h or 18 h. For clarity, the fluorescent (shown in red) and transmission images were superimposed and shown in the left panels. The fluorescence images alone are also shown in white and black (right panels) for better visualization of the signal analyzed. Scale bars: 10 µm.

(B) Box plot of the number of fluorescent dots per area for control HL-1 cells and HL-1 cells either exposed to 2 or 18 h to nocodazole as shown in *A*. Numbers below each box represent the number of cells analyzed. Each box represents the IQR (25th and 75th quartile) with the median indicated by a line. Whiskers above and below indicate the 95% confidence interval. Statistical significance (One-Way ANOVA, ***$p \leq 0.001$; ** $p \leq 0.01$). Despite the changes in Golgi fragmentation induced by nocodazole, HL-1 cells treated with the same nocodazole regime showed no alterations of the L-type current density amplitudes and voltage dependence of activation compared to control cells (Main text Figure 2).

Figure S2

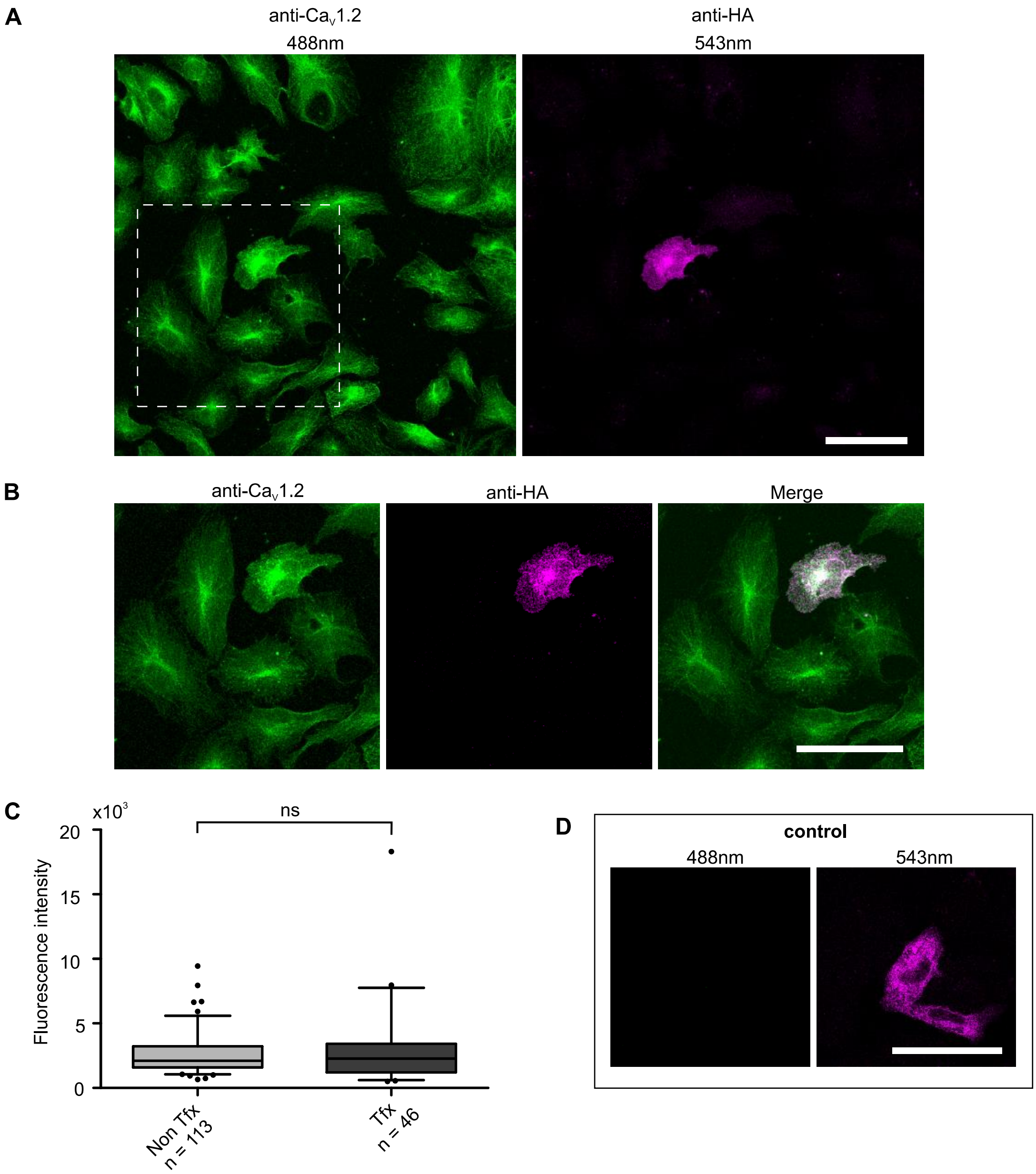

Figure S2. Comparable expression levels of Ca$_V$1.2 in transfected and untransfected HL-1 cells, Related to Figure 3.

(A) Laser scanning confocal images of HL-1 cells transfected with Ca$_V$1.2-HA- and Ca$_V$β-subunit-encoding plasmids and immunostained for Ca$_V$1.2 (green) and HA (magenta).

(B) Enlarged view of the marked square region including a representative transfected cell.

(C) Box plot of the fluorescence intensity from anti-CaV1.2 labeled channels measured in entire cells either expressing CaV1.2-HA (Tfx n = 46) or non-expressing (non Tfx n = 113).

(D) Control laser scanning confocal images of HL-1 cells transfected with Ca$_V$1.2-HA- and Ca$_V$β-subunit-encoding plasmids and immunostained for only HA-Tag (magenta). No fluorescence of the HA-Tag is visible by exciting with the 488 nm laser.

Significance tested with t-test with Welch‘s correction, p = 0.05. Scale bars: 50µm.

Figure S3

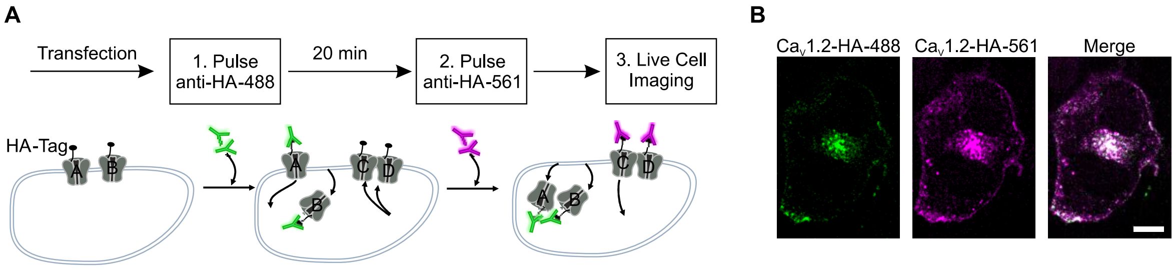

Figure S3. Fluorescence images of HL-1 cells expressing extracellularly HA-tagged Ca_V1.2 exposed to a dual-pulse labeling protocol demonstrate channel turnover within a few tens of minutes, Related to Figure 3.

(A) Dual-pulse labelling protocol combined with spinning disk confocal microscopy was used to visualize newly-inserted Ca_V1.2 channels in HL-1 cells. HL-1 cells were transfected with a cDNA encoding for Ca_V1.2-HA channels and for Ca_Vβ. Two consecutive labelling reactions, with anti-HA-DyLight 488 (step1, shown in green) and DyLight 561 (step2, shown in magenta), were performed 20 min apart and cells were immediately imaged after the second pulse (step 3). The scheme shows channels stained during the first antibody pulse (Ca_V1.2-HA-488), denoted with A and B, and during the second antibody pulse (Ca_V1.2-HA-561), denoted with C and D. Only Ca_V1.2 channels inserted after the first pulse (C and D) can be stained during the second pulse and represent the newly-inserted channel pool.

(B) Representative HL-1 cell showing Ca_V1.2 channels stained during anti-HA-488 and anti-HA-561 antibody pulses in green and in magenta, respectively. The merge image is shown in the right panel with overlapping pixels in white. Scale bar: 10 µm.

Figure S4

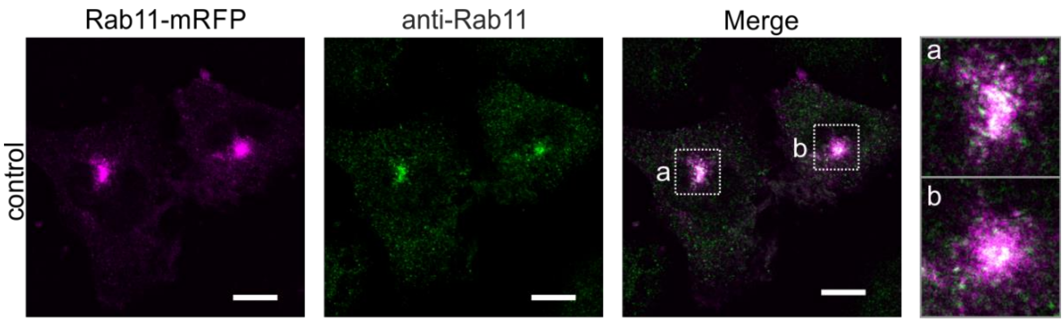

Figure S4. Immunodetected Rab11a colocalizes with heterologously expressed Rab11a-mRFP in HL-1 cells, Related to Figure 5.

Laser scanning confocal images of HL-1 cells expressing Rab11a-mRFP (magenta) and immunostained with anti-Rab11a antibody (green). Overlapping pixels appear in white in the merged figure. Panels at the right show an enlarged view of the corresponding marked square regions from the merged image. Scale bar: 10 µm.

Figure S5

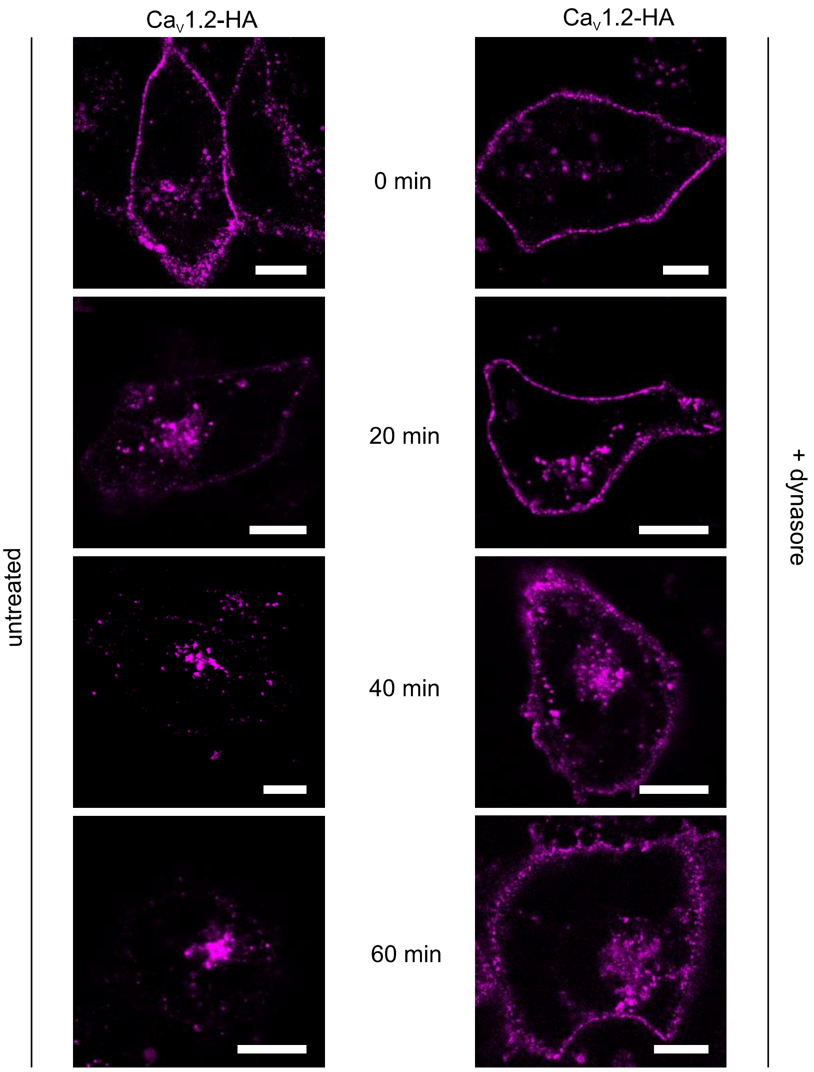

Figure S5. $Ca_V1.2$-HA channels exhibit longer dwelling times at the plasma membrane of dynasore-treated HL-1 cells than of untreated cells, Related to Figure 5.

Laser scanning confocal images of HL-1 cells expressing HA-tagged $Ca_V1.2$ and $Ca_V\beta$. Cells were stained with anti-HA-DyLight 561 and visualized at different time points after the staining procedure (0, 20, 40, 60 min). Cells were either untreated (left images) or treated with 80 µM dynasore one hour before and during the staining process (right images). Dynasore-treated cells show a prominent membrane staining up to 60 min, while in untreated cells the membrane staining disappeared after 20 min. Scale bar: 10µm

Figure S6

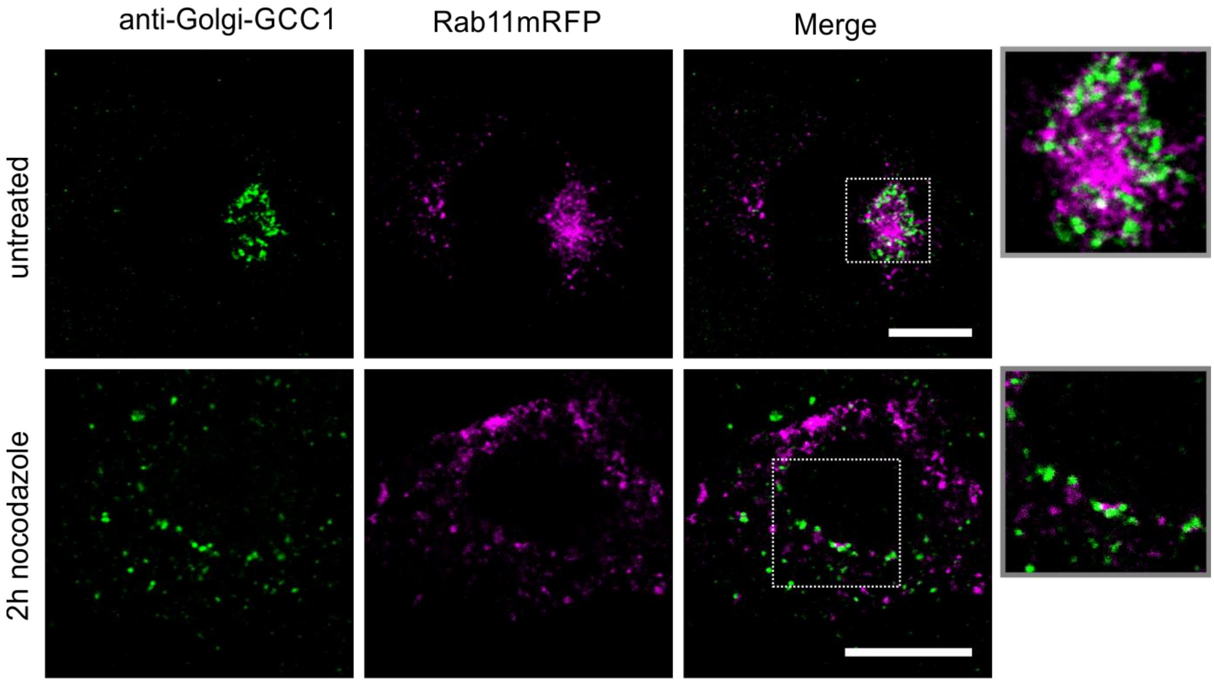

Figure S6. Nocodazole treatment does not result in random overlapping of dispersed fragments from Golgi and Rab11a-endocytic recycling compartment, Related to Figure 5.

Laser scanning confocal images of HL-1 cells expressing Rab11a-mRFP (magenta) and stained with anti-Golgi-GCC1 antibody (green) in untreated cells and after exposing the cells for 2 h to nocodazole. Overlapping pixels appear in white in the merge figures. Right panels correspond to the enlargement of the corresponding marked squares in the merged images. Scale bars: 10 µm.

Transparent Methods

cDNA constructs. The plasmid encoding $Ca_V1.2$-HA was generated by inserting the HA-Tag into the external II-III loop of the rabbit $Ca_V1.2$ pore-forming alpha subunit (accession number UniProtKB P15381) into the pcDNA3.1(+) vector. The coding region of the rat β-subunit (accession number UniProtKB Q8VGC3-2) was subcloned in pcDNA3.1(+) vector by standard techniques. The plasmid encoding the rat lysosomal membrane glycoprotein 1 (Lamp1) fused to the monomeric red fluorescent protein (mRFP) (Lamp1-mRFP) was a gift from Walther Mothes (Addgene plasmid # 1817). The coding regions of Rab7 (Addgene plasmid # 61803) and Rab11 (a gift from Richard Pagano (Addgene plasmid # 12605) were subcloned by standard techniques into mRFP vector (pmRFP) to express Rab proteins coupled to mRFP as described (Guzman et al., 2015).

Cell culture and transfection. The murine atrial cardiomyocyte cell line HL-1 (a kind gift from Dr. W.C. Claycomb) was grown in Claycomb media supplemented with 10% fetal bovine serum (SigmaAldrich), 10 µM norepinephrine (SigmaAldrich), 2 mM L-glutamine (SigmaAldrich) and 100 U/ml penicillin/streptomycin (ThermoFisher Scientific) at 37°C in 5% CO_2 as described (Stölting et al. 2015). Exchange of media was done every 24–48 h. HL-1 cells were seeded 1–2 days prior to performing the corresponding experiment on fibronectin precoated 18 mm glass coverslips for laser scanning confocal microscopy on immunostained fixed cells, 35 mm dishes (ibidi) for live cell imaging using spinning disk microscopy, 8-well plates (ibidi) for single molecule localization microscopy and on 25 mm glass coverslips for electrophysiology.
Cells were transfected with $Ca_V1.2$- and $Ca_V\beta_{2a}$-encoding plasmids (2:1) (Figure 3) and the same mixture with or without Rab11a- or Lamp1-encoding plasmids (Figure 6) using Lipofectamine 2000 (ThermoFisher Scientific). Live cell imaging was performed 24 h after transfection in phenol-red-free DMEM media (ThermoFischer Scientific).

Pharmacological treatment. To disrupt actin filaments, HL-1 cells were incubated for 2 h in medium (DMEM) containing 10 µM cytochalasin D (SigmaAldrich) at 37°C in 5% CO_2. Afterwards the medium was replaced and cells were washed with PBS and fixed with 4% paraformaldehyde (SigmaAldrich). For the disruption of microtubules, HL-1 cells were incubated for the indicated time periods in medium (DMEM) containing 33 µM nocodazole (SigmaAldrich) at 37°C in 5% CO_2 and fixed as described above. To inhibit endocytosis, cells were treated for 2 h with 80 µM dynasore (SigmaAldrich) before fixation.

Cell fixation and immunostaining. The following antibodies were used at the indicated dilutions: rabbit anti-$Ca_V1.2$-ATTO-488 (1:200, Alomone labs), mouse anti-β-tubulin (1:500,

ThermoFisher Scientific), mouse anti-Clathrin (1:100; BD Bioscience), mouse anti-Rab11-D3 (1:250, Santa Cruz), mouse anti-Rab7-B3 (1:250; Santa Cruz) and rabbit anti-Golgi-GCC1 (1:500, BD Bioscience). The fluorophore-conjugated secondary antibodies donkey anti-mouse antibody coupled to Cy3 or Cy5 (1:200, Dianova) or donkey anti-rabbit-Cy3 (1:200, Dianova) was used. Actin filaments were stained with phalloidin coupled to Alexa Fluor 647 (1:500; Invitrogen). HL-1 cells were fixed with 4% paraformaldehyde (PFA; SigmaAldrich) in PBS onto a 37°C heat plate for 5 minutes. All following steps were done at room temperature. Fixed cells were simultaneously permeabilized, blocked and incubated with the corresponding primary antibody using 5% chemiBLOCKER (Merck) supplemented with 0.05% Triton-X100 for 1 h. Cells were then washed twice with PBS and stained with the appropriate secondary antibody for 30 minutes in 5% chemiBLOCKER-0.05% Triton. After washing the cells (twice with PBS) coverslips were mounted on glass slides using Aqua Poly Mount (Polysciences) and stored at 4°C until use.

Immunofluorescent staining of living cells. Transiently transfected HL-1 cells with Ca_V1.2-HA were washed with ice cold PBS and stained with mouse anti-HA Tag DyLight 561 or DyLight 488 (1:100, ThermoFisher) in media (OptiMEM) for 10 minutes at room temperature. Immediately after washing, the cells were mounted on the microscope Stage Top Chamber (Okolab) incubator at 37°C for live cell imaging using spinning disk confocal microscopy.
For the pulse chase experiment, HL-1 cells were first stained with the mouse anti-HA Tag DyLight 488 as above, incubated for 20 minutes at 37°C and 5% CO_2 and stained again using mouse anti-HA Tag DyLight 561. Following the washing step, cells were immediately mounted in the spinning disk confocal microscope for live cell imaging.

Laser scanning confocal fluorescence microscopy. Confocal imaging was carried out on a Leica inverted confocal microscope using a 63x/1.4 NA oil immersion objective (Nikon). To visualize Atto488 fluorescence, cells were excited with a 488nm argon laser. For Cy3 and mRFP fluorescence a 543 nm excitation HeNe laser was used. Phalloidin coupled to Alexa Fluor 647 was imaged using a 633 nm excitation laser. For presentation purposes, images were exported, formatted and brightness adjusted using Fiji ImageJ (Schindelin et al., 2012). The emitted light was monitored between 480–540 nm, 574–625 nm and 650–795 nm, respectively

Spinning disk confocal fluorescence microscopy. For all experiments, cells were mounted inside the StageTop Incubator system (Okolab) on the microscope stage for precise control of the temperature (37°C), humidity (85%), and CO_2-concentration (5%). The spinning disk confocal microscope was equipped with a confocal laser scanning microscope (Nikon Eclipse

Ti) combined with a Confocal Scanner Unit (CSU-W1, Yokogawa). Using an optical beam splitter, the excitation at 488 nm and 561 nm is measured simultaneously and the emitted light was collected between 500-550 nm and 590-650 nm, respectively. Images were acquired with a 100x/1.49 oil immersion objective at 512x512 pixels. The software Andor IQ2 was used for acquisition. For all live-cell time-lapse recordings the exposure time was set to 200 ms and the frame rate to the minimal. The laser power and the number of focal planes (z-frames) were set individually for every measured cell.

Analysis of the colocalization via Manders Overlap Coefficient. Colocalization between two different fluorescent proteins was evaluated using the intensity correlation coefficient-based (ICCB) analysis to evaluate the Manders overlap coefficient embedded in the PlugIn "JACoP" in the ImageJ software (Bolte and Cordelieres, 2006). Here, only the fraction of Ca_V1.2–fluorescence overlapping the respective fluorophores in the compartment are reported.

Quantification of cell surface Ca_V1.2 and time constant of internalization. The levels of cell surface expression of Ca_V1.2 under different pharmacological treatment conditions were estimated by quantifying the fluorescence intensity of the plasma membrane using ImageJ. For each analyzed cell, two regions of interest (ROIs) adjacent to the cell boundary were drawn manually: one around the whole cell including the plasma membrane (whole cell) and a second one at the cell periphery (cell interior), including the cytoplasm of the cell but excluding the cell boundary. The fluorescence of the cell interior, measured as 'integrated density', was subtracted from that of the whole cell to obtain the fluorescence intensity signal at the plasma membrane (surface Ca_V1.2). The shown values were normalized to the area of the plasma membrane of each analyzed cell. The time constant of channel internalization was calculated as the decay of fluorescence intensity at the cell surface. Using ImageJ, 6 to 15 ROIs per cell in different z-planes, were drawn manually around fractions of the plasma membrane. The fluorescence in every ROI was measured as 'integrated density' over time, treated as an independent measurement and individually fitted to a mono exponential decay. Scaling and offset parameters from the fits were used to linearly transform each curve, such that all start at 1 and decay to 0. All measurements that yielded nonsensical fit parameters, i.e. negative value for one of the parameters, were excluded from the analysis leaving n = 109 datasets for further investigations. Mean and SD of all the transformed fit lines to the raw data are shown in Figure 3D. The reported time constant was obtained by performing a global fit to a mono-exponential decay to all linearly transformed curves. The uncertainty of the fitted time constant was estimated via bootstrap sampling (Efron and J.Tibshirani, 1993) where 109 (number of ROIs) random samples with replacements are taken from the original set of 109 measurements

and a global fit to this synthetic data was performed. This procedure was repeated 50 000 times and the reported time constant value is given by the mean value and SD of those 50 000 time constants. The bootstrapped time constant distribution and the mean time course of the individual fits to the bootstrap samples are shown in Figure 3E.

Single Molecule Localization Microscopy (SMLM). Fixed HL-1 were washed and permeabilized by incubation in PBS supplemented with 0.5% Triton X-100 for 10 min and blocked for 45 min with 5% normal goat serum (SigmaAldrich) dissolved in PBS. Cells were then incubated for 1 h with the corresponding primary antibody diluted in the same blocking solution followed by several washes with PBS supplemented with 0.1% Tween-20. The cells were incubated with the secondary antibody for detecting tubulin (diluted in blocking buffer), washed twice with PBS supplemented with 0.1% Tween-20 and stored at 4°C until the next day for single molecule localization super-resolution microscopy analysis. Single-molecule localization microscopy data acquisition and analysis was done on our custom-built widefield/TIRF microscope based on an Olympus IX-71 inverted microscope body, as previously described (Stölting et al., 2015). Images were recorded at 80 nm/px in widefield mode with an EMCCD camera (Andor iXon DU897E-C00-#BV) cooled to −75°C. Acquisition was performed at room temperature using imaging buffer (50 mM β−mercaptoethylamine in degassed PBS). Detection of tubulin (Alexa 647) using excitation at 642 nm was followed by measurement of $Ca_V1.2$ (ATTO 488) with excitation at 488 nm. The obtained data was corrected for chromatic aberration. Single molecule localizations were determined from the data using SNSMIL (Tang et al., 2015).

Electrophysiology. Whole-cell patch-clamp technique was used to record calcium currents 24–72 h after cell splitting with a HEKA EPC 10 amplifier using HEKA´s Patchmaster software, as described (Stölting et al., 2015). Recording solutions were as follows: extracellular solution contained in mM: 157 TEA-Cl, 5 $CaCl_2$, 0.5 $MgCl_2$ and 10 HEPES, pH 7.4; internal solution: 125 CsCl, 20 TEA-Cl, 3.6 PCr-Na_2, 10 EGTA, 5 Mg-ATP, 0.2 Na-GTP and 10 HEPES, pH 7.4. HL-1 cells were held at a holding potential of −40 mV and L-type calcium currents were elicited by 100 ms voltage pulses ranging from the holding potential to +60 mV in 10 mV increments.

Statistical Analysis. Statistical analysis and comparisons between the groups were conducted using the GraphPad Prism 5 software. Statistical differences between the data sets were analyzed using One-Way ANOVA followed by post hoc Bonferroni's Multiple Comparison test ($p \leq 0.001$). The data are presented as box plots representing the interquartile range (IQR) with median, 25th and 75th percentile. The whiskers indicate the 95% confidence interval of

the data. Outliers are shown by dots. N is the number of cells given above or below each box in all graphs.

References

Bolte, S., and Cordelieres, F.P. (2006). A guided tour into subcellular colocalization analysis in light microscopy. Journal of microscopy *224*, 213-232.

Efron, B., and Tibshirani, R. J. (1993). An Introduction to the Bootstrap. Monographs on Statistics and Applied Probability. Chapman & Hall/CRC, Boca Raton, Florida, USA.

Guzman, R.E., Miranda-Laferte, E., Franzen, A., and Fahlke, C. (2015). Neuronal ClC-3 Splice Variants Differ in Subcellular Localizations, but Mediate Identical Transport Functions. The Journal of biological chemistry *290*, 25851-25862.

Schindelin, J., Arganda-Carreras, I., Frise, E., Kaynig, V., Longair, M., Pietzsch, T., Preibisch, S., Rueden, C., Saalfeld, S., Schmid, B., Tinevez, J.Y., White, D.J., Hartenstein, V., Eliceiri, K., Tomancak, P., and Cardona, A. (2012). Fiji: an open-source platform for biological-image analysis. Nature methods *9*, 676-682.

Stölting, G., de Oliveira, R.C., Guzman, R.E., Miranda-Laferte, E., Conrad, R., Jordan, N., Schmidt, S., Hendriks, J., Gensch, T., and Hidalgo, P. (2015). Direct interaction of $Ca_V\beta$ with actin up-regulates L-type calcium currents in HL-1 cardiomyocytes. The Journal of biological chemistry *290*, 4561-4572.

Tang, Y., L. Dai, X. Zhang, J. Li, J. Hendriks, X. Fan, N. Gruteser, A. Meisenberg, A. Baumann, A. Katranidis, and T. Gensch. (2015). SNSMIL, a real-time single molecule identification and localization algorithm for super-resolution fluorescence microscopy. *Scientific reports*. 5:11073.
